# Noninvasive cerebral oxygenation monitoring during rapid ventricular pacing in transcutaneous aortic valve implant

**DOI:** 10.1186/cc10897

**Published:** 2012-03-20

**Authors:** J Dens, I Meex, F Jans, H Gutherman, C De Deyne

**Affiliations:** 1 Ziekenhuis Oost-Limburg, Genk, Belgium

## Introduction

Most recent attention in interventional cardiology is now directed towards treatment of valvular heart disease. In patients with high-risk cardiac surgery, transcutaneous aortic valve implantation (TAVI) could offer a therapeutic solution. Near-infrared spectroscopy (NIRS) has been introduced as a useful noninvasive cerebral monitoring technique assessing cerebral oxygenation. As of today, no reports have been published on the use of any NIRS technology during TAVI procedures. During valve prosthesis implantation, a cardiac standstill by rapid ventricular pacing (RVP) is induced to minimize cardiac motion. While RVP is advantageous for valve positioning, a combination of rapid heart rate and ventricular hypertrophy can induce a complete loss of cardiac output. In most cases, this hemodynamic deficit is well tolerated, due to the brief duration of RVP. But as of today no data are available on cerebral oxygenation during these critical periods of RVP.

## Methods

We report on 10 consecutive patients (>75 years, major comorbidities) suffering from severe aortic stenosis. Bilateral ForeSight sensors were applied after induction of anesthesia. We were especially interested if any change in cerebral oxygenation (SctO_2 _monitoring) occurred during these RVP periods.

## Results

In all patients, the procedure was technically successfully performed. Mean SctO_2 _before RVP was 67% (59 to 71%) and immediately decreased during RVP to mean 54% (37 to 70%). In seven patients, RVP resulted in SctO_2 _decreases below 55% (mean 44%; range 37 to 52%). These decreases lasted for mean 20 minutes (14 seconds to 87 minutes). Systolic blood pressure before RVP was mean 135 mmHg (95 to 165 mmHg) and decreased to mean 74 mmHg (112 to 42 mmHg) during RVP. In six patients, RVP resulted in a decrease in systolic blood pressure below 90 mmHg, which was immediately countered by vasoactive drugs (adrenaline). In two patients, extensive hypotension persisted despite vasoactive support and CPR had to be initiated. In one patient, SctO_2 _values remained below 55% for 87 minutes and the patient was declared brain dead 48 hours later.

## Conclusion

Transcutaneous cardiac interventions, especially those with transient cardiac standstill, can induce longlasting intraprocedural inadequacy of cerebral perfusion, despite immediate restoration of normal blood pressure. Future strategies should therefore be focused on optimalizing cerebral oxygenation before RVP.

